# Case series: The value of fundus autofluorescence in inherited macular disease

**DOI:** 10.1002/ovs2.70036

**Published:** 2026-04-22

**Authors:** Marina Guro, Henrietta Wang, Jack Phu, Nimesh B. Patel, Kaitlyn A. Sapoznik, Hamza Shah, Michael Kalloniatis

**Affiliations:** ^1^ University of Houston College of Optometry Houston Texas USA; ^2^ School of Optometry and Vision Science University of New South Wales Sydney New South Wales Australia; ^3^ Centre for Eye Health Sydney New South Wales Australia; ^4^ Faculty of Medicine and Health University of Sydney Sydney New South Wales Australia; ^5^ Concord Repatriation General Hospital Concord Hospital Sydney New South Wales Australia; ^6^ School of Medicine (Optometry) Deakin University Waurn Ponds Victoria Australia

## Abstract

**Purpose:**

To evaluate the diagnostic utility of fundus autofluorescence (FAF) imaging in identifying and characterizing phenotypically classified inherited macular dystrophies. In this way, we aim to provide methods by which eye care practitioners can link FAF imaging and other clinical results or imaging modalities to aid their clinical decision‐making.

**Case Reports:**

Phenotypically identified inherited macular dystrophies, including Stargardt disease and related ABCA4 mutations, Best vitelliform dystrophy, pattern dystrophies, and cone and cone‐rod dystrophies, are discussed.

**Conclusions:**

We provide evidence that the use of FAF alone and in combination with other clinical results and imaging modalities can assist in the diagnosis of a range of inherited macular dystrophies.

## Significance

The diagnosis of inherited macular disease is complex, particularly in older populations where confounding conditions such as age‐related macular degeneration are prevalent. Moreover, younger individuals may exhibit minimal retinal changes, which complicates diagnostic decision‐making. Having a method that can help discriminate inherited macular disease from non‐inherited macular disease or from those with normal retinae would be useful in clinical practice.

## INTRODUCTION

The term “inherited macular diseases” refers to a genetically heterogeneous group of progressive disorders affecting central photoreceptors and retinal pigment epithelium (RPE) cells. The group includes different types of dystrophies: ABCA4 gene mutations (Stargardt disease and ABCA4‐associated cone‐rod dystrophy), cone and cone‐rod dystrophies not associated with ABCA4 mutations, Best vitelliform dystrophy, pattern dystrophies, Sorsby fundus dystrophy, and autosomal dominant drusen.^1^, ^2^, ^3^

Fundus autofluorescence (FAF) is a noninvasive diagnostic tool that is useful in the diagnosis of different retinal disorders, including age‐related macular degeneration (AMD),^4^, ^5^ central serous chorioretinopathy,^6^, ^7^ and inherited retinal dystrophies.^8^, ^9^ FAF utilizing short‐wavelength blue light is most commonly used in a clinical setting, which enables the detection of lipofuscin in the RPE.^9^ Another type of FAF is based on near‐infrared wavelength light, which can detect an additional source of autofluorescence, melanin, and allows deeper penetration into the posterior eye, providing further information about RPE disturbances and better visualization of the fovea and parafoveal region.^8^ Near‐infrared FAF shows melanin‐containing structures as hyperautofluorescent. For this reason, the fovea appears hyperautofluorescent, in contrast to short‐wavelength FAF. Hyperpigmented structures also appear hyperautofluorescent.^10^ Melanin has antioxidant properties and protects photoreceptors against oxidative stress associated with the accumulation of lipofuscin. Individuals with reduced melanin content in the RPE are more susceptible to the development of AMD, and near‐infrared FAF may be useful for AMD prediction and monitoring.^8^

Lipofuscin is the main retinal fluorophore detected in short‐wavelength FAF. It is a mixture of by‐products called bisretinoids, formed as a result of vitamin A metabolism and other processes occurring within the visual cycle.^11^ After bisretinoids have been formed in photoreceptors, they are captured by RPE cells and accumulate within lysosomes.^8^ N‐Retinyl‐N‐retinylidene ethanolamine (A2E) is the most well‐studied component of lipofuscin. A2E is a lysosome‐resistant substance, as it has an inhibitory effect on protein and glycosaminoglycan catabolic pathways in the RPE lysosomes.^12^ A2E and other metabolic by‐products of vitamin A accumulate in RPE cells and exert toxic effects on them, leading to RPE cell loss.^8^, ^11^

Another pathological mechanism of fundus fluorophore formation is the disruption of RPE phagocytosis of shed photoreceptor outer segments. In this case, the accumulation of fluorophores occurs in the subretinal space, which is a characteristic, for example, of Best vitelliform dystrophy.^8^, ^13^

FAF is an important tool in the diagnosis of inherited macular diseases. It helps with differential diagnosis and, in many cases, enables early disease detection. In the early stages of inherited macular diseases, many diagnostic methods—color fundus photography (CFP) or variations thereof (such as OPTOMAP imaging), optical coherence tomography (OCT), and visual field testing (VF)—typically reveal nonspecific pathological changes.^14^, ^15^ Although electrophysiological investigations are often very useful,^16^ the availability and length of time necessary for testing often limit their applicability in clinical practice. Under normal conditions, fluorophores are present in all healthy RPE cells, allowing the normal retina the ability of autofluorescence. Lipofuscin accumulates in the RPE with normal aging, but excessive accumulation can occur in retinal diseases.^17^ FAF enables noninvasive detection of areas with normal autofluorescence, areas of pathological fluorophore accumulation, and RPE loss. Pathological fluorophore accumulation is seen as hyperautofluorescence, whereas RPE cell loss is seen as hypoautofluorescence. On short‐wavelength FAF under nonpathologic conditions, the fovea appears hypoautofluorescent due to the presence of lutein, zeaxanthin, and melanin, which block autofluorescence of lipofuscin.^9^ In contrast, in the early stages of inherited macular diseases, especially cone dystrophies, the fovea can appear hyperautofluorescent due to lipofuscin accumulation, which may sometimes be the only specific sign of this disease group.^18^

Many modern fundus cameras are equipped with FAF imaging technology and offer the ability to assist clinicians in the diagnosis of various retinal diseases by detecting pathological lipofuscin accumulation and obscured RPE cell loss. FAF assists in distinguishing between different conditions; for example, in young patients, it is a valuable diagnostic method for inherited macular diseases, whereas in older individuals, it aids in the differential diagnosis between inherited and acquired macular diseases. The examination does not require any special preparation or intravenous administration of contrast agents and takes very little time to perform. In this case series, we describe examples of the following disorders: ABCA4‐associated dystrophies, Best vitelliform dystrophy, pattern dystrophies, and other nonspecified cone dystrophies and cone‐rod dystrophies. The purpose of this case series is to highlight the usefulness of FAF, both in the early and advanced stages of inherited macular diseases, and to encourage clinicians to use this diagnostic tool in their everyday practice.

## METHODS

In this study, clinical records from the Centre for Eye Health (CFEH), Sydney, were reviewed. CFEH is an optometry‐led care clinic that provides enhanced access to modern imaging and diagnostic technologies for patients at high risk for various ophthalmic diseases.^19^, ^20^ In compliance with the Centre for Eye Health protocols, all reports were reviewed by senior clinicians with arranged ophthalmology consultations (general or a subspecialty ophthalmologist from the local health district), if needed. After completing the review, the final diagnosis was determined using clinically available results. Subjects with diagnoses of likely inherited macular disease were chosen for this case series from the database. Only short‐wavelength FAF scans were used in this study. Additionally, only one eye is shown if the pathological changes in both eyes were similar. All patients provided written consent for use of their clinical data for research purposes, in accordance with the Declaration of Helsinki, and this study was approved by a Biomedical Human Research Ethics Advisory Panel of the University of New South Wales, Australia.

Instruments used to collect images for this case series were the fundus retinal camera (Kowa WX 3D non‐mydriatic retinal camera, Kowa, Tokyo, Japan, https://ophthalmic.kowapharma.com/products/retinal‐cameras/nonmyd‐wx3d‐simultaneous‐stereoscopic‐photography‐retinal‐camera‐specs/), Optomap ultra‐widefield with fundus autofluorescence imaging (Optomap Panoramic 200Tx, Optos, Dunfermline, Scotland, UK, https://www.optos.com/products/), OCT (Spectralis HRA2 + OCT, Heidelberg Engineering, Heidelberg, Germany, https://www.heidelbergengineering.com/en‐US/products/spectralis; Cirrus HD‐OCT, Carl Zeiss Meditec, Dublin, CA, USA, https://www.zeiss.com/meditec/en/products/optical‐coherence‐tomography‐devices.html), and standard automated perimetry using the Humphrey field analyzer (Carl Zeiss Meditec, Dublin, CA, USA, https://www.zeiss.com/meditec/en/products/perimetry.html).

## CASE REPORTS

**FIGURE 1:**
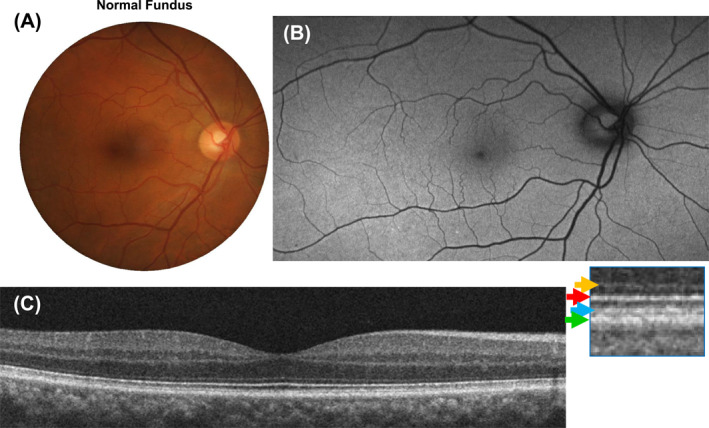
A 58‐year‐old male with no ocular pathology. (A) Color fundus photograph of the posterior pole. (B) FAF showing the normal appearance of autofluorescence with a slight reduction centrally. (C) OCT of the central macular region. The four hyperreflective outer retinal bands are visible: external limiting membrane (orange), ellipsoid zone (red), interdigitation zone (blue), and RPE (green) in the inset. The distal outer two layers are discriminated mostly in the central region of 1C. FAF, fundus autofluorescence; OCT, optical coherence tomography.

Normal fundus findings are presented at the beginning of the case series. Figure 1 shows the normal right eye, CFP and FAF (Figure 1A,B), of a 58‐year‐old male showing the normal reduction in FAF in the foveal region. The OCT image shows a normally appearing structure with the inset showing four highly reflective bands of the outer retina depicting the external limiting membrane, ellipsoid zone, interdigitation zone, and the RPE^21^ (Figure 1C).

### ABCA4 mutation

The first group includes the ABCA4‐associated dystrophies (Stargardt disease, fundus flavimaculatus, and ABCA4‐associated cone‐rod dystrophy). These disorders are caused by ABCA4 mutations and are inherited in an autosomal recessive or an autosomal dominant mode of inheritance.^3^

The ABCA4 gene is part of the ATP‐binding cassette family, which controls active cellular membrane transport. In this disease group, the main pathological mechanism is the dysfunction of ABCA4, resulting in the disruption of membrane transport of retinoids during the visual cycle.^2^, ^11^ Impairment of the normal transport of retinoids leads to toxic by‐product (including lipofuscin) accumulation. This process has a harmful effect on photoreceptors and RPE and leads to their gradual degeneration and loss. The phenotype in ABCA4 dystrophy depends on the degree of ABCA4 suppression.^2^, ^8^

ABCA4‐associated dystrophies typically present with a gradual decline in central vision starting in childhood or adolescence. In addition to central vision loss, patients can experience difficulties with dark adaptation, color vision impairment, and reduced contrast sensitivity. Fundoscopy findings at early stages usually include yellow‐white flecks across the posterior pole; the RPE in the macula shows signs of degeneration and atrophy, often described as a “beaten bronze” or “bull's eye” pattern. RPE atrophy can progress with time and extend to involve nearly the entire macula or the entire retina.^3^, ^22^ Hyperautofluorescent flecks at the perifoveal zone and along the vascular arcades can be appreciated on FAF. In some cases, the fovea can exhibit increased autofluorescence or may appear mottled, with nonhomogeneous hyperautofluorescent and hypoautofluorescent patterns. In advanced stages, areas of RPE atrophy can be seen as dark or hypoautofluorescent lesions on FAF. OCT can show loss of photoreceptors, ellipsoid zone degenerative changes, hyperreflective material accumulation, and RPE atrophy.^2^, ^21^ An interesting finding in this type of dystrophy is that the area around the optic nerve remains unaffected, appearing normal on fundus examination and on FAF.^22^, ^23^, ^24^

#### Case 1

**FIGURE 2:**
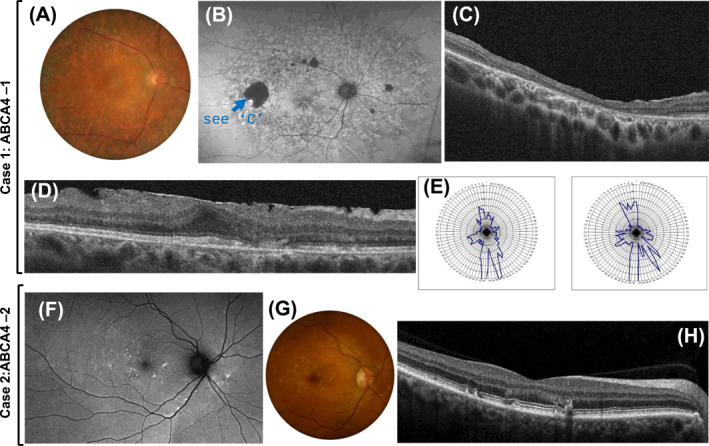
Two cases typical of ABCA4 mutations. (A–E) are from a 64‐year‐old female displaying classic features present in the ABCA4 mutation. (A) CFP; (B) FAF; (C) OCT image from the large, degenerated region in the temporal retina of the right eye (identified in (B)); (D) central OCT showing the impact of the ERM; (E) 100 hue color vision test with a classic tritan defect OU. (F–H) are from a 56‐year‐old with a classic fundus flavimaculatus appearance. (F) FAF; (G) CFP; (H) OCT image through the right fovea. For both cases, the left eye displayed similar imaging results. CFP, color fundus photography; ERM, epiretinal membrane; FAF, fundus autofluorescence; OCT, optical coherence tomography.

Figure 2A–E shows a 64‐year‐old female with visual acuities of 20/30 OD and 20/40 OS with classic features of advanced ABCA4 mutation and an epiretinal membrane (ERM) (both eyes were similar). The CFP displayed a mottled appearance and evidence of an ERM. The FAF showed areas of hyperautofluorescence and hypoautofluorescence and a normal appearance around the optic nerve. Figure 2C is an OCT scan through the large area of RPE loss (dark region temporal to the optic nerve in panel 2b) displaying extensive retinal degeneration. The OCT image (Figure 2D) displays the distortion of the macular region due to the ERM. Panel 2e shows a classic tritan defect on the FM‐100 hue test,^25^ which is expected in these conditions.^26^

#### Case 2

The second case is a 56‐year‐old female with visual acuities of 20/20 OU and lesions surrounding her macular region that have changed over the years, consistent with fundus flavimaculatus. CFP shows fleck‐like yellow spots surrounding the macular region (Figure 2G), more prevalent in the right eye. These regions corresponded to mixed hyperautofluorescent and hypoautofluorescent patterns (Figure 2F) with corresponding anomalies evident in the OCT images (Figure 2H).

In both cases, FAF imaging provided evidence of RPE changes and lipofuscin accumulation, which add important information for the diagnosis.

### Best vitelliform dystrophy

In most instances, Best vitelliform dystrophy is an autosomal dominant condition. Nevertheless, several cases of autosomal recessive inheritance have been reported.^27^, ^28^ Best vitelliform dystrophy is associated with the BEST1 gene (previously named VMD2) mutation, which leads to dysfunction of a protein called bestrophin 1. This protein regulates calcium‐activated chloride channels and voltage‐dependent calcium channels within the RPE cell membranes. Proper functioning of bestrophin1 is essential for the efficient processing of waste products, such as lipofuscin, by RPE cells.^13^, ^29^

In Best vitelliform dystrophy, dysfunction of RPE phagocytosis leads to lipofuscin accumulation in the RPE, the subretinal space, and the photoreceptor zone. Additionally, the BEST1 mutation leads to cholesterol homeostasis dysregulation in the RPE and Bruch's membrane.^29^ The disease typically begins in childhood or adolescence. The fundus abnormalities progress through several distinct stages. The first stage is the previtelliform stage with a near‐normal macular appearance. OCT findings are unremarkable or can show some hyperreflective material accumulation under the fovea at the interdigitation zone and RPE layers. In turn, FAF can show abnormal hyperautofluorescence in the fovea even at the previtelliform stage, that is, before vitelliform material accumulation is evident.^30^ The vitelliform stage is presented by a single egg yolk‐like lesion in the fovea (the vitelliform lesion). OCT imaging shows vitelliform material accumulation between the interdigitation zone and the RPE. The vitelliform lesion is seen as a hyperautofluorescent lesion in the fovea on FAF. The next stage of the disease is often called the “pseudohypopyon” stage as vitelliform material undergoes disorganization and resorption. Fundoscopy shows vitelliform material migration to the lower portion of the vitelliform lesion, which corresponds to hyperautofluorescence in the lower part of the lesion and hypoautofluorescence in the upper part. In some cases, advanced stages of Best vitelliform dystrophy can be complicated by choroidal neovascularization (CNV). The last stage of the pathological process in Best vitelliform dystrophy is atrophy or fibrotic tissue formation if CNV was present.^31^, ^32^ FAF offers useful insights into vitelliform lesions throughout the various stages, with increased autofluorescence early in the disease process and hypoautofluorescence after atrophy. Widefield FAF additionally provides an overview of extramacular changes that occur.^30^

#### Case 3

**FIGURE 3:**
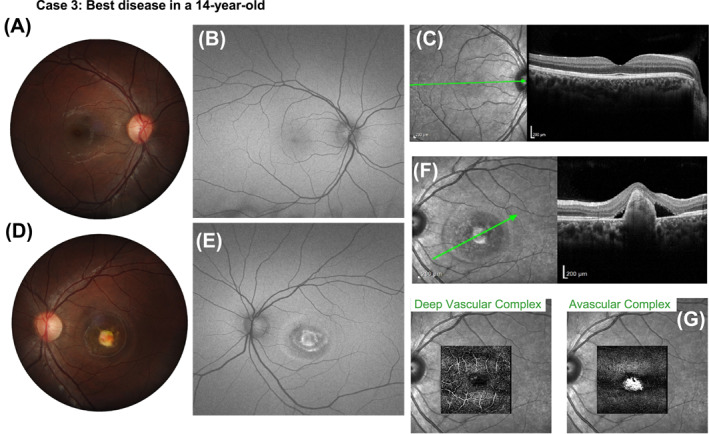
Best disease in a 14‐year‐old male. The (A) CFP, (B) FAF, and (C) OCT are unremarkable in the right eye. The left eye shows a large yellow area on (D) CFP, hyperautofluorescent lesion on (E), and a confirmed vitelliform lesion on OCT (F). OCT angiography of the deep vascular complex was normal (left panel of (G)), but a dense neovascular membrane is present in the deep avascular zone (right panel of (G)). CFP, color fundus photography; FAF, fundus autofluorescence; OCT, optical coherence tomography.

This case (Figure 3) is that of a 14‐year‐old male with visual acuities of 20/15 OD and 20/50 OS with Best vitelliform dystrophy in the left eye. CFP, FAF, and OCT of the right eye were normal (Figure 3A–C). The left eye showed a round yellow lesion in the fovea (Figure 3D), corresponding to the area of hyperautofluorescence (Figure 3E), with OCT imaging showing hyperreflective sub‐RPE material accumulation surrounded by neurosensory detachment (Figure 3F), indicating the vitelliform stage (advanced stage). OCT‐A displays a neovascular membrane under the vitelliform lesion within the avascular zone (Figure 3G).

#### Case 4

**FIGURE 4:**
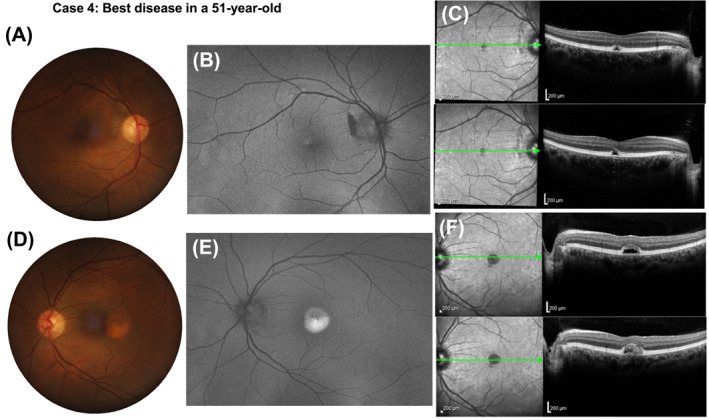
Best disease in an older individual, the 51‐year‐old father of *Case 3*. He denied vision disturbance beyond the need for reading spectacles despite an obvious reduction in visual acuity of his left eye. The right eye CFP (A) is unremarkable; however, the FAF shows an area of hyperautofluorescence at the fovea typical of a previtelliform lesion (B). This area reflects the focal subretinal hyporeflective space with disruption and elevation of the ellipsoid zone evident in the two OCT images (C). The left eye has a large yellow hyperautofluorescent vitelliform lesion evident in all three imaging modalities (D–F). CFP, color fundus photography; FAF, fundus autofluorescence; OCT, optical coherence tomography.

This case (Figure 4) is that of the father of the patient in Case 3, who showed a vitelliform‐like lesion in the left eye. The presence of the vitelliform lesion in both father and son likely indicates a dominant inheritance pattern. The 51‐year‐old father had a long‐standing history of poor vision in his left eye, with presenting acuities of 20/40 OD and 20/50 OS. The CFP of the right eye displayed a near‐normal macula, with FAF showing a slightly hyperautofluorescent foveal appearance (Figure 4A,B). The right eye OCT image displayed a focal subretinal hyporeflective space with disruption and elevation of the ellipsoid zone (Figure 4C). The left eye had a large vitelliform lesion (large yellow lesion) evident in the CFP and seen as a large hyperautofluorescent image slightly inferiorly (Figure 4D,E). OCT imaging showed hyperreflective material inferiorly and serous detachment superiorly (Figure 4F). There was no evidence of CNV in either eye after OCT‐A imaging. These findings are consistent with the previtelliform stage in the right eye and the pseudohypopyon stage in the left eye. The large vitelliform lesion, almost the size of the optic nerve head, is characteristic of Best disease rather than adult‐onset vitelliform dystrophy (Figure 4D–F).

The cases above highlight the appearance of Best disease at different ages, the usefulness of FAF in the previtelliform stage, and the importance of testing for CNV.

Pattern dystrophies are a group of polymorphic autosomal dominant macular diseases. This group includes adult‐onset foveomacular vitelliform dystrophy, butterfly‐shaped pigment dystrophy, reticular dystrophy, multifocal pattern dystrophy simulating Stargardt disease, and fundus pulverulentus. The genotype range for these disorders is very broad, with mutations in various genes (PRPH2, IMPG1, IMPG2, CTNNA1, OTX2, BEST1, and PYGM). The main feature in the pathogenesis of these disorders is dysfunction of RPE waste product processing and, in turn, lipofuscin accumulation.^33^, ^34^

### Pattern dystrophies

Adult‐onset foveomacular vitelliform dystrophy is a condition that has many similarities to Best vitelliform dystrophy. Several gene mutations, including PRPH2, BEST1, IMPG1, and IMPG2, are known to cause this disorder. Unlike Best vitelliform dystrophy, adult‐onset foveomacular vitelliform dystrophy typically presents after the age of 40. It is characterized by slow progression and preserved visual acuity for an extended period of time if the vitelliform lesion is stable. The disease can progress to the pseudohypopyon stage or can be complicated by CNV formation, which leads to vision deterioration, but CNV is not a universal finding.^35^ It is important to distinguish adult‐onset foveomacular vitelliform dystrophy from age‐related macular dystrophy to avoid unnecessary treatment. The main feature of this disease is the formation of a yellowish vitelliform lesion, most commonly a single round focus in the fovea without neurosensory retinal edema. This lesion can be seen on OCT as hyperreflective material at the RPE‐interdigitation zone level, presenting as bright hyperautofluorescence on FAF. The intensity of the vitelliform lesion autofluorescence is greater than the hyperautofluorescence of drusen in AMD.^4^ This dystrophy can undergo the same stages as Best vitelliform dystrophy, and in that case, OCT and FAF show the same pattern as in Best vitelliform dystrophy.^36^

#### Case 5

**FIGURE 5:**
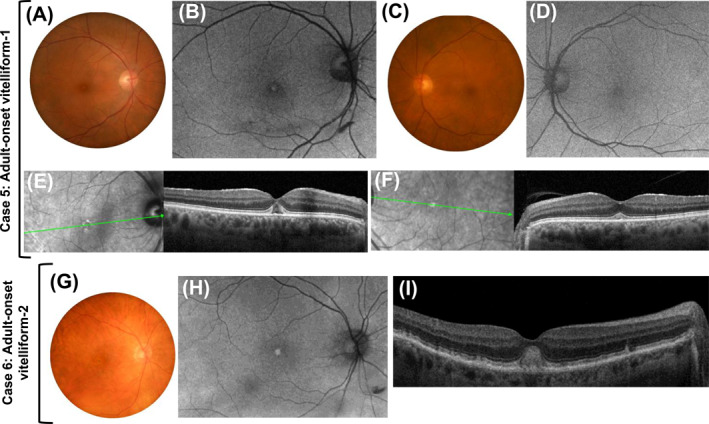
Adult‐onset foveomacular vitelliform dystrophy in a 77‐year‐old patient. The CFP (A, C) shows a yellowish area in the right eye but no noticeable abnormality in the left eye. Both FAF images display slightly elevated foveal autofluorescence (OD > OS) (B, D). The OCT images show vitelliform lesions in both foveae (E, F). Panels (G–I) are from a 90‐year‐old patient with minimal CFP changes but a clear area of hyperautofluorescence (H), with the OCT showing a vitelliform lesion with multiple drusen within the posterior pole. CFP, color fundus photography; FAF, fundus autofluorescence; OCT, optical coherence tomography.

This 77‐year‐old male was referred to the Centre for a macular assessment with slightly reduced visual acuity of 20/30. The CFP showed a small yellowish area in the right eye and no obvious change in the left eye (Figure 5A,C). The FAF showed an area of hyperautofluorescence in the right eye with a slight increase in autofluorescence centrally in the left eye (Figure 5B,D). The OCT images showed an early ERM in both eyes, bilateral subfoveal subretinal thickening at the interdigitation zone, and vitelliform hyperreflective material OU with slight liquefaction in the right eye (Figure 5E,F). There was no CNV detected in either eye after OCT‐A.

#### Case 6

A 90‐year‐old male with visual acuities of 20/40 OD and 20/75 OS was referred for a macular assessment. CFP was relatively unremarkable, with an area of hyperautofluorescence under FAF and a subfoveal vitelliform lesion, as well as mixed cuticular and reticular pseudodrusen in both eyes on OCT (Figure 5G–I). OCT‐A did not show CNV in either eye.

The fundus findings in both cases may be mistaken for features of AMD, such as subfoveal drusen, RPE dystrophy, or fibrotic lesions. In this situation, FAF helps in the differential diagnosis and to determine patient management.

### Reticular retinal dystrophies

Reticular retinal dystrophy is characterized by specific fundus changes such as pigment redistribution in a reticular pattern and irregularly distributed yellowish deposits, which can be present in the macula or outside the macula. Visual acuity varies from relatively preserved in the case of paramacular changes^37^ to significantly reduced when the macula is involved.^38^ OCT shows RPE dystrophic changes and deposits of hyperreflective material at the RPE level. FAF presents with a reticular pattern of hyperautofluorescence and hypoautofluorescence.^38^, ^39^

#### Case 7

**FIGURE 6:**
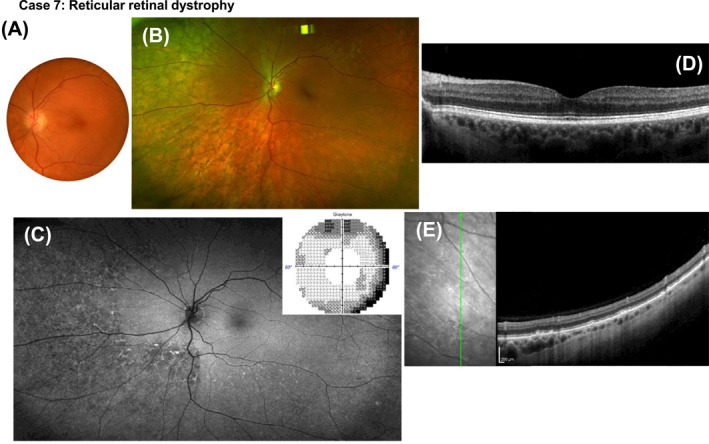
A 65‐year‐old patient with reticular retinal dystrophy in both eyes. CFP shows some change at the edge of the image (A), but widefield imaging (B) and FAF (C) highlight the anomalies in more peripheral locations. Central OCT appears normal (D), with pigment clumping and RPE anomalies evident on OCT images in affected locations (E). The 60‐4 test grid grayscale showing midperipheral visual field disturbance is inserted into panel (C). CFP, color fundus photography; FAF, fundus autofluorescence; OCT, optical coherence tomography.

A 65‐year‐old female was referred to the Centre for a general assessment with visual acuities of 20/20 OU. She avoided driving at night and knew of her decline in midperipheral vision (confirmed with visual field assessment: Figure 6C inset). There was reticular pigment clumping in the nasal midperiphery, more evident in the widefield images (Figure 6A–C). FAF showed a reticular pattern of hyperautofluorescence and hypoautofluorescence outside the macula (Figure 6C). The OCT images presented a normal appearance centrally (Figure 6D) with pigment deposits corresponding to small bumps in the RPE in more peripheral locations (Figure 6E). These findings are typical of reticular retinal dystrophy. The 60‐4 test grid displayed midperipheral visual field loss (inserted in panel 6C). Although this patient did not have significant alterations in the macular region, we included this case in our series to highlight reticular changes on FAF that are pathognomonic for reticular retinal dystrophy.

Butterfly‐shaped pattern dystrophy has an autosomal dominant mechanism of inheritance and can be caused by peripherin/RDS (PRPH2) gene mutations.^40^ The condition is typically diagnosed in adulthood, although cases of pathological changes in the fundus have been documented in childhood.^39^ The course of the disease is relatively benign. Most individuals maintain high visual acuity, normal color vision, and normal electroretinogram findings despite reduced electro‐oculography values over several decades.^40^ However, in some cases, absolute scotomata may develop in the central and paracentral visual fields as the disease progresses.^41^

The disease is characterized by the appearance of a butterfly‐shaped pattern of hypopigmentation or hyperpigmentation in the macula. Peripapillary chorioretinal atrophy and progressive macular dyspigmentation are also typical features as the disease advances. FAF shows hypoautofluorescent spots in combination with a butterfly‐shaped hyperautofluorescent pattern in the macula, corresponding to hyperreflective material accumulation evident on OCT.^42^, ^43^

#### Case 8

**FIGURE 7:**
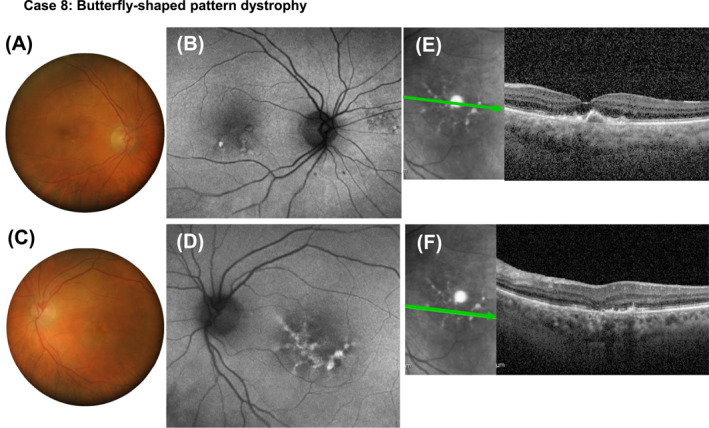
Butterfly‐shaped pattern dystrophy in an 87‐year‐old female with a history of reduced vision. CFP (A, C) shows hypopigmented areas; however, the butterfly‐shaped pattern is found on FAF (B, D). Anomalies in the outer retina are evident in the OCT images (E, F) that include drusen‐like anomalies and disruption of the outer retinal morphology. CFP, color fundus photography; FAF, fundus autofluorescence; OCT, optical coherence tomography.

An 87‐year‐old female was referred to the Centre for a macular assessment with a history of reduced vision. Visual acuity testing revealed 20/50 OD and 20/100 OS. CFP showed hypopigmentation in the macular region, and FAF showed a butterfly‐shaped pattern in both eyes, but more prominent in the left eye (Figure 7A–D). There were basal laminar drusen‐like abnormalities, attenuation/loss of the interdigitation zone, and reticular pseudodrusen in both eyes (Figure 7E,F). There was no evidence of CNV in either eye after OCT‐A imaging. The butterfly‐shaped pattern observed on FAF is atypical for AMD and assists in the differential diagnosis with the age‐related ocular disease. Thus, without FAF, this condition could be confused with AMD.

Multifocal pattern dystrophy simulating STGD1/fundus flavimaculatus is associated with peripherin/RDS (PRPH2) mutations. Similar to ABCA4 Stargardt disease, this dystrophy is characterized by the formation of yellowish deposits in the posterior pole in combination with areas of RPE atrophy. Patients may be asymptomatic or present with decreased visual acuity after the fifth decade of life. FAF shows hyperautofluorescent foci corresponding to the yellowish deposits, along with areas of hypoautofluorescence representing zones of RPE atrophy.^43^

#### Case 9

**FIGURE 8:**
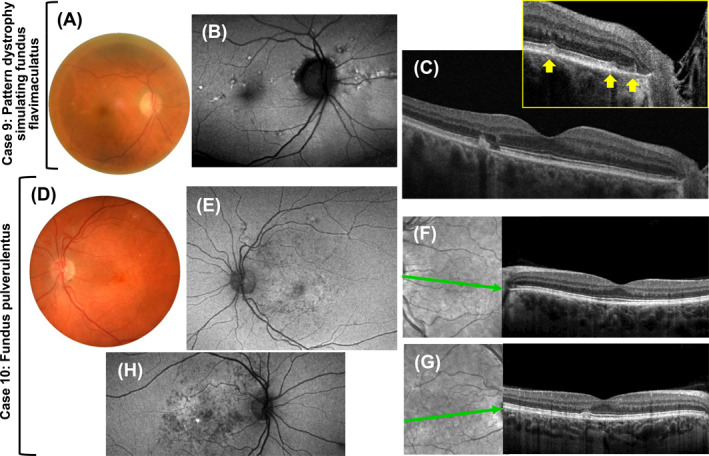
Multifocal pattern dystrophy simulating fundus flavimaculatus (A–C) and fundus pulverulentus (D–H). The CFP (A) shows areas of fleck‐like anomalies that correspond to intense hyperautofluorescence in FAF imaging (B). These same areas show disruption on OCT imaging associated with hyperreflectivity ((C) and inset: yellow arrows point to lesions close to the optic nerve). In the fundus pulverulentus case, the CFP (D) is unremarkable, and the FAF images (E, H) show significant hypoautofluorescence and hyperautofluorescence. The right FAF image has a small vitelliform‐like lesion evident in both the FAF (H) and OCT image (G). The OCT imaging shows disruption at the RPE location and choroidal hypertransmission (F, G). CFP, color fundus photography; FAF, fundus autofluorescence; OCT, optical coherence tomography.

A 70‐year‐old female was referred to the Centre for a macular assessment. Her entering visual acuities were 20/25 OD and 20/70 OS. The CFP showed flecks located at the maculae and extending beyond the posterior poles, clearly evident in the FAF image (Figure 8A,B). OCT images showed areas of hyporeflective and hyperreflective lesions that extended into the photoreceptor layer and corresponded to FAF changes. Remarkably, the peripapillary zone was involved in the pathological process and had the same hyperreflective lesions (Figure 8B), which should not be present in the case of true ABCA4 dystrophy (Figure 8C inset shows an OCT with lesions next to the optic nerve). Based on the FAF and OCT findings, the condition was diagnosed as multifocal pattern dystrophy simulating fundus flavimaculatus.

Fundus pulverulentus is a rare pattern dystrophy characterized by pigment redistribution in the macula and focal accumulation of hyperreflective material in the macular area seen on OCT. In some cases, it is accompanied by a subfoveal neurosensory retinal detachment in the fovea, with or without the presence of subretinal CNV.^44^, ^45^ FAF reveals multifocal punctate hypoautofluorescence in combination with areas of hyperautofluorescence.^45^

#### Case 10

A 48‐year‐old male was referred to the Centre for a macular assessment, with visual acuities of 20/15 OU. The CFP was unremarkable, with the FAF image showing hypoautofluorescent abnormalities with speckled hyperautofluorescence centrally and a small focal hyperautofluorescent vitelliform lesion inferotemporally in the right eye (Figure 8G–H). OCT findings showed mild hyperreflective disruptions of the RPE and choroidal hypertransmission with no evidence of retinal atrophy (Figure 8F,G). These findings are most consistent with fundus pulverulentus, a variant of pattern dystrophy.

### Cone and cone‐rod dystrophies

The group of cone dystrophies and cone‐rod dystrophies includes two subgroups: primarily stationary and progressive cone/cone‐rod dystrophies. The primarily stationary subgroup includes autosomal recessive achromatopsia (CNGA3, CNGB3, and GNAT2 gene mutation) and X‐linked hereditary blue cone monochromasia (X‐linked gene mutation). These mutations cause a stationary congenital severe color vision deficiency.^46^

The subgroup of progressive cone/cone‐rod dystrophies is genetically and phenotypically heterogeneous, as there are mutations in more than 30 genes that were reported to cause cone/cone‐rod dystrophies. These genes control many different processes in the retina, such as phototransduction cascades, outer segment formation, intraflagellar transport, and neurotransmitter release.^47^, ^48^

These dystrophies are characterized by early onset and, in many cases, fast deterioration of central and color vision. X‐linked opsin gene mutations are among the best studied in this group of diseases. The phenotype varies based on the type of mutation. Patients from the same family may have different rates of vision loss and varying degrees of fundus changes.^49^ Individuals with X‐linked red‐green color vision deficiency caused by L/M opsin mutations may have cone dystrophy in addition to the red‐green color vision deficiency: They show progressive thinning of the outer nuclear layer and reduced cone density, leading to decreased central vision.^50^

Other common symptoms are photophobia and central and paracentral scotomata. Overall, the clinical presentation and the appearance of the fundus are typically polymorphic. Fundus changes are often nonspecific and can vary in severity, from mild pigment redistribution to significant areas of RPE atrophy. OCT shows disruption of the interdigitation zone and progressive loss of photoreceptors. Zones of RPE atrophy can be present in advanced stages.^47^ FAF may present with either hyperautofluorescence or hypoautofluorescence in the fovea and macula, depending on the mutation type and stage of the disease. FAF patterns can vary from mottled hyperautofluorescent and hypoautofluorescent appearances to large areas of hypoautofluorescence due to RPE cell loss. At early stages, the fovea often appears hyperautofluorescent, but as the disease progresses and areas of RPE atrophy develop, hypoautofluorescence starts appearing on FAF.^18^

#### Case 11

**FIGURE 9:**
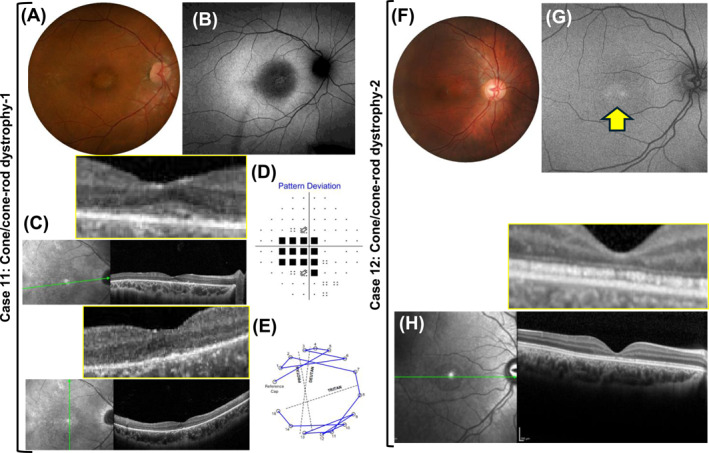
Cone/cone‐rod dystrophies in a 15‐year‐old (A–E) and a 10‐year‐old (F–H). There is a “bull's eye” appearance in the CFP (A) and a large ring of hypoautofluorescence surrounding the fovea and an outer ring of hyperautofluorescence further out. The autofluorescence in the fovea is slightly elevated (B). The OCT imaging (C) shows central outer retinal degeneration with associated visual function loss (D) and an acquired blue‐yellow color vision deficiency (E). Panel (C) has inserts of the foveal regions. The CFP in the 10‐year‐old is unremarkable (F), and the FAF shows a central area of hyperautofluorescence (arrow in (G)). A “bull's eye” appearance is evident in the infrared image of the OCT (H), and the granulation of the outer retinal layers indicative of central dystrophy is clearly evident (inset in (H) is of the foveal area). CFP, color fundus photography; FAF, fundus autofluorescence; OCT, optical coherence tomography.

A 15‐year‐old male was referred to the Centre for an assessment due to chronic bilateral reduction of visual acuity and unusual foveal reflex. Visual acuities were 20/40 OD and 20/30 OS. CFP showed a “bull's eye” macular appearance, with the FAF showing paramacular hyperautofluorescence, perifoveal hypoautofluorescence, and a hyperautofluorescent fovea (Figure 9A,B). On OCT there was a small subfoveal ellipsoid zone island remaining, with loss of parafoveal and perifoveal ellipsoid zones, with the four outer retinal regions noted in Figure 1 missing (Figure 9C). Midperipheral and peripheral OCT appeared normal OU. Both eyes displayed central loss on 30‐2 visual field testing (Figure 9D from the right eye), and a tritan defect was evident using L'Anthony desaturated D‐15 (Figure 9E). Both eyes displayed similar imaging and functional results.

#### Case 12

A 10‐year‐old male was referred for electrophysiological testing due to chronic decreased visual acuity of 20/70 OU. CFP was unremarkable; however, FAF imaging showed an area of hyperautofluorescence in the macular region (Figure 9F,G). OCT imaging showed foveal thinning and granularity in the ellipsoid‐interdigitation zone, with the infrared image showing a “bull's eye” appearance on infrared imaging (Figure 9H). The left eye imaging results were similar. Both the rod and cone flash ERG responses were slightly delayed but within expected amplitude limits. Cone flicker was abnormal in amplitude and showed a longer implicit time OS and only a longer implicit time in OD.

#### Case 13

**FIGURE 10:**
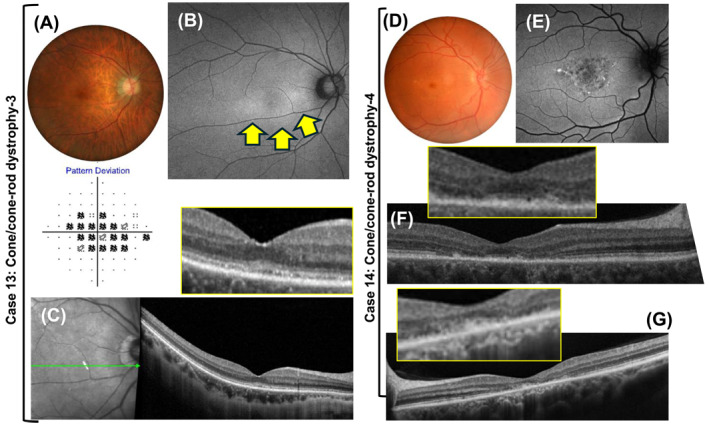
A 65‐year‐old female with a history of slight vision reduction over the years. The CFP (A) is unremarkable, but careful assessment of the FAF image shows macular hyperautofluorescence (arrows in (B)). OCT imaging (C) shows granulation in the central region (inset in (C)) indicative of a retinal dystrophy and an associated functional loss using the 10‐2 central visual field (visual field inset in (C)). A 47‐year‐old male with reduced vision in the left eye first noticed it two years earlier. There are yellowish deposits and depigmentation in the CFP (D) and speckled hyperautofluorescence and hypoautofluorescence in FAF (E). There is overall central thinning of the retina with degeneration of the outer retina present in both eyes (F, G, with inserts of the foveal region), with a small central area in the right eye (F) that is relatively spared, showing a likely external limiting membrane (inset). CFP, color fundus photography; FAF, fundus autofluorescence; OCT, optical coherence tomography.

A 65‐year‐old female with a history of visual disturbance was referred to the Centre for a macular assessment. Visual acuities were 20/40 OD and 20/50 OS. CFP was unremarkable; however, FAF imaging showed a parafoveal hyperautofluorescence in both eyes (Figure 10A,B). OCT imaging showed a granular appearance of the ellipsoid zone/interdigitation zone (Figure 10C with inset). The 10‐2 visual field test grid showed central depression in both eyes (inset in 10C is from the right eye). Electrophysiological responses showed normal rod responses with cone amplitudes just outside the normative ranges for both eyes. Panel D‐15 results showed a deutan defect in both eyes.

#### Case 14

A 47‐year‐old male was referred to the Centre for a retinal dystrophy assessment. He first noticed changes in his vision 2 years earlier with entering acuities of 20/25 OD and 20/200 OS. There were yellow deposits and depigmentation in both maculae evident in the CFP, with the FAF images showing a mixed speckled hyperautofluorescence and hypoautofluorescence that corresponded to the fundoscopic macular changes (Figure 10D,E). OCT showed central outer retinal atrophy (worse in the left eye) with central sparing evident in the right eye (Figure 10F,G).

## CONCLUSIONS

The multitude of genotypes leads to diverse phenotypes in inherited macular diseases, complicating the diagnosis, particularly in elderly patients. The diagnosis of this group of dystrophies is a complex process that requires the systematic use of various diagnostic methods. In the case of macular pathology, the results of functional and imaging tests should be evaluated using a holistic approach to create a full clinical picture. The tests should be sensitive to detect specific changes and relevant in terms of recognizing the pathological patterns associated with the presumed disease. A pattern can be common for several disease groups, but the degree of manifestation and combination with the other test results can create a holistic picture. For example, in older patients, fundus changes in AMD and inherited macular diseases may look similar. However, FAF patterns differ in these two disease groups. Although pathological changes in AMD, such as drusen, can be represented in patchy, linear, and reticular patterns, they typically show less bright hyperautofluorescence compared to inherited macular dystrophy cases. The intensity of FAF (e.g., bright foci of hyperautofluorescence in ABCA4 mutations) and specific patterns of hyperautofluorescent distribution (such as a single foveolar focus in adult‐onset foveomacular vitelliform dystrophy, a butterfly‐shaped pattern in butterfly‐shaped pattern dystrophy, or “bull's eye” patterns in ABCA4 dystrophies), in combination with OCT findings, help establish the correct diagnosis.^4^

Geographic atrophy shows a hypoautofluorescent pattern in the RPE atrophy zone, which is common for any case of RPE cell loss, with various phenotypic patterns of autofluorescence at the junctional zone of progression—some of which are related to higher rates of atrophy enlargement.^4^, ^51^ Central serous chorioretinopathy, depending on the stage of the disease, may present with blocked FAF, mottled FAF, hyperautofluorescent or hypoautofluorescent patterns, and descending tracts.^7^

FAF has the advantage of being a noninvasive fast assessment of the retina with unique patterns for different inherited macular diseases (summarized in Table 1). The systematic review of different FAF studies, conducted by Frampton et al.,^52^ showed 55%–100% sensitivity and up to 100% specificity rates depending on the condition being assessed.

**TABLE 1:** A summary of FAF patterns for the inherited macular diseases.

Disease	FAF pattern
ABCA4 mutation	‐ Hyperautofluorescent flecks at the perifoveal zone and along the vascular arcades; ‐ Increased autofluorescence in the fovea or a mottled appearance in the macula, with nonhomogeneous hyperautofluorescent and hypoautofluorescent patterns (“bull's eye” pattern); ‐ In advanced stages, areas of RPE atrophy can be seen as dark lesions with mottled hyperautofluorescence perifocally; ‐ The area around the optic nerve remains unaffected even in the advanced stage of the disease.
Best vitelliform dystrophy	‐ Early stage: Increased autofluorescence in the fovea; ‐ Vitelliform stage: Round bright hyperautofluorescent lesion in the fovea; ‐ Pseudohypopyon stage: Round lesion with a level and increased hyperautofluorescence in the inferior half of the lesion; ‐ Late atrophic stage: Single focal or multiple confluent areas of hypoautofluorescence; ‐ Size of vitelliform lesion: A large‐sized lesion compared to that found in adult‐onset vitelliform dystrophy.
Adult‐onset foveomacular vitelliform dystrophy	‐ The same stages as in Best vitelliform dystrophy, but the bright hyperautofluorescent lesion in the fovea is usually smaller than in Best vitelliform dystrophy. In some cases, several vitelliform lesions may be present.
Reticular retinal dystrophy	‐ Reticular pattern of hyperautofluorescence and hypoautofluorescence, mostly seen outside the macular region.
Butterfly‐shaped pattern dystrophy	‐ Hypoautofluorescent foci in combination with a butterfly‐shaped hyperautofluorescent pattern in the macula.
Multifocal pattern dystrophy simulating STGD1/fundus flavimaculatus	‐ Hyperautofluorescence along with areas of hypoautofluorescence. It can involve the area around the optic nerve.
Fundus pulverulentus	‐ Multifocal punctate hypoautofluorescence in combination with areas of hyperautofluorescence.
Cone dystrophies, cone‐rod dystrophies	‐ At early stages, the fovea often appears hyperautofluorescent with a “bull's eye” appearance commonly seen; ‐ As the disease progresses and areas of RPE atrophy develop, hypoautofluorescence starts appearing on FAF; FAF patterns can vary from a mottled hyperautofluorescent and hypoautofluorescent appearance and a “bull's eye” pattern to large areas of hypoautofluorescence.

Abbreviations: FAF, fundus autofluorescence; RPE, retinal pigment epithelium.

FAF is a valuable tool, as changes in autofluorescence indirectly provide information about RPE function in terms of accumulation of waste products, such as lipofuscin, or loss of RPE cells. This method aids in differential diagnosis, and in some cases, FAF abnormalities can be the most specific signs of the dystrophy, displaying early signs of ocular disease. Moreover, FAF is an important tool in the differential diagnosis between inherited macular diseases and other macular pathologies. The use of this method in routine clinical practice may help improve diagnostic accuracy, prevent misdiagnoses, and decrease the number of unnecessary treatments.

## AUTHOR CONTRIBUTIONS

**Marina Guro**: Conceptualization; data curation; writing—original draft; writing—review and editing. **Henrietta Wang**: Data curation; writing—review and editing. **Jack Phu**: Conceptualization; data curation; writing—review and editing. **Nimesh B. Patel**: Data curation; writing—review and editing. **Kaitlyn A. Sapoznik**: Writing—review and editing. **Hamza Shah**: Writing—review and editing. **Michael Kalloniatis**: Conceptualization; data curation; writing—original draft, writing—review and editing.

## CONFLICT OF INTEREST STATEMENT

The authors declare no conflicts of interest.
